# Persistence of potential ST398 MSSA in outpatient settings among US veterans, 2010–2019

**DOI:** 10.1017/ash.2023.452

**Published:** 2023-10-20

**Authors:** Margaret Carrel, Qianyi Shi, Shinya Hasegawa, Gosia S. Clore, Michael Z. David, Eli N. Perencevich, Matthew Smith, Michihiko Goto

**Affiliations:** 1 Department of Geographical & Sustainability Sciences, University of Iowa, Iowa City, IA, USA; 2 Department of Internal Medicine, University of Iowa, Iowa City, IA, USA; 3 Center for Access & Delivery Research and Evaluation (CADRE), Iowa City Veterans Affairs Health Care System, Iowa City, IA, USA; 4 Department of Medicine, University of Pennsylvania, Philadelphia, PA, USA

## Abstract

Novel ST398 methicillin susceptible *Staphylococcus aureus* (MSSA) in the United States was first observed in New York City (2004**–**2007); its diffusion across the country resulted in changing treatment options. Utilizing outpatient antimicrobial susceptibility data from the Veterans Health Administration from 2010 to 2019, the spatiotemporal prevalence of potential ST398 MSSA is documented.

## Introduction

Sequence type 398 (ST398) *Staphylococcus aureus* emerged as a novel type globally in the early 2000s.^1,2^ ST398 with methicillin resistance (MRSA) was commonly associated with livestock contact, while ST398 with methicillin susceptibility (MSSA) was not.^3^ Genomic analysis indicates further contrasts between livestock-associated ST398 MRSA and human-associated ST398 MSSA, such as the varying presence of genes encoding resistance to tetracycline and erythromycin.^2,4^ Although ST398 MSSA was widely detected in European and Asian countries, in the United States it was first detected in New York City (NYC) and the subsequent region of greatest prevalence was New York state and the Northeast.^5–8^ Utilizing the phenotypic characteristics commonly associated with ST398 MSSA, prior analysis of MSSA collected nationwide in US hospitals documented an increased prevalence of potential ST398 from 2003 to 2014 with a significant negative association with distance from NYC.^8,9^ Molecular characterization of MSSA isolates across the United States from 2004 to 2010 similarly found a low prevalence of this phenotype outside of the Northeast.^10^

This phenotypic profile has been shown to be associated, but perhaps not exclusively, with ST398 MSSA. However, in the absence of widespread genotyping of *S. aureus* isolates, spatiotemporal surveillance of phenotypic profiles can indicate the prevalence and diffusion patterns of novel antimicrobial resistance varieties. Utilizing a retrospective cohort of all *S. aureus* positive cultures in outpatient Veterans Health Administration (VHA) settings from 2010 to 2019, we explored whether previously observed rates of potential-ST398 MSSA held steady or changed over time and whether the Northeast continued to serve as the epicenter for this phenotype.

## Data and methods

All *S. aureus* isolates from clinical specimens collected in outpatient VHA settings from January 1, 2010 to December 31, 2019 were analyzed. Microbiology reports were extracted from the Corporate Data Warehouse, the integrated electronic medical record system data repository of the VHA health system. We included all isolates from patients with a residential address in the contiguous United States who were ≥18 yr old at the time of specimen collection. Isolates were excluded from analysis if they were obtained at institutionalized care settings (e.g., acute care, nursing homes, rehabilitation facilities) or if they were taken after either 48 h of an inpatient admission or 72 h of an inpatient discharge. MSSA was defined as susceptibility to first-generation cephalosporins, cefoxitin, nafcillin, oxacillin, or carbapenems. Only isolates that were tested for resistance against all three relevant antimicrobial classes, clindamycin, erythromycin, and tetracycline, were included in the analysis. The first record per unique patient per month was retained. Potential ST398 was defined as resistance, including intermediate resistance, to clindamycin and erythromycin with susceptibility to tetracycline.^7,8^ When reported, inducible clindamycin resistance (commonly known as D-test) was also considered to be resistance.

Based on patient county of residence, MSSA isolates were assigned to Census regions: Northeast, Midwest, South, or West. Prevalence of potential ST398 MSSA was determined at county and regional scales for each month and year of the study.

With the county-level monthly counts of potential ST398 as the outcome variable, a Poisson regression model with robust standard errors was fitted to account for the variation in the trends of potential ST398 between 2010 and 2019 as repeated measurements within each region. Region was included in the model as a fixed effect and an interaction term between time and region was included in the model to detect regional variability in temporal trends. The total number of MSSA infections was included as an offset variable. Baseline incidence rates and temporal trends were estimated as incidence rate ratios (IRRs), using Northeast and the year 2010 as references.

The study was approved by the IRB of the University of Iowa.

## Results

**Figure 1.**
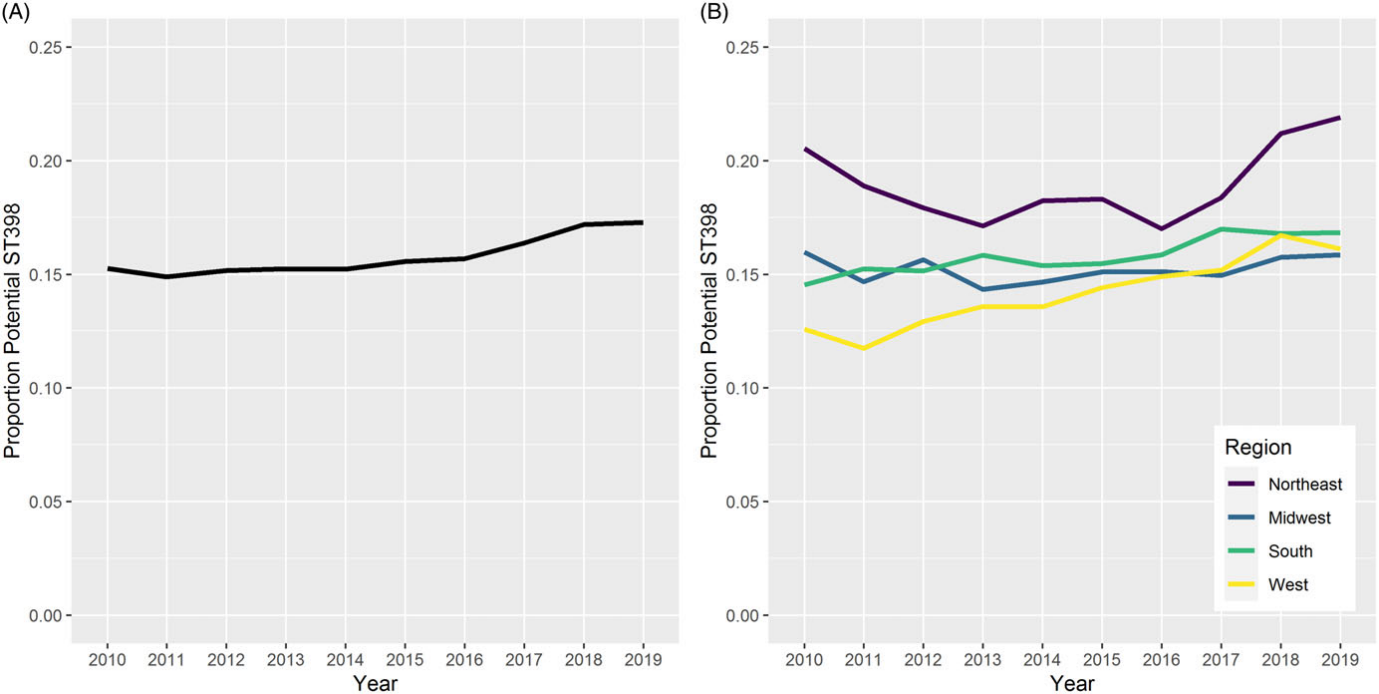
(A) Proportion of MSSA isolates with clindamycin and erythromycin resistance and tetracycline susceptibility, potential ST398, in outpatients from 2010 to 2019; (B) proportion of MSSA isolates that are potential ST398 by Census region.

In 2010**–**2019, 235,033 outpatient specimens were positive for MSSA and 161,816 (68.85%) were tested against all three relevant antimicrobial classes. Among the first 145,304 isolates per unique patient per month, 22,919 (15.77%) were classified as potential ST398. Rates of potential ST398 rose slightly from ∼15% in the early years of the study to ∼17% in the final years (Figure 1A). Prevalence differed by Census region, with the Northeast having the highest prevalence of potential ST398 and the West having the greatest increases in rates over time (Figure 1B).

Models accounting for region and time showed similar patterns of potential ST398 MSSA (Table 1). No significant change in prevalence over time was observed (IRR: 1.01 [0.99**–**1.02]). The Midwest, South, and West all had significantly lower potential ST398 MSSA than did the Northeast. When the interaction between time and region was examined, only the West had significantly increased potential ST398 from 2010 to 2019 (IRR: 1.02 [1.01**–**1.05]) compared to the Northeast.

**Table 1.** Incidence rate ratios (IRR) for MSSA isolates with clindamycin and erythromycin resistance and tetracycline susceptibility, potential ST398, in outpatients over time and by region and with interaction between region and year

	IRR (95% CI)	Robust std. err.
(Intercept)	0.18 (0.17, 0.19)	0.01
Year	1.01 (0.99, 1.02)	0.01
Northeast	–	–
Midwest	**0.83 (0.77, 0.** **91)**	**0.04**
South	**0.82 (0.76, 0.88)**	**0.03**
West	**0.67 (0.61, 0.72)**	**0.03**
Year:Midwest	0.99 (0.97, 1.01)	0.01
Year:South	1.00 (0.99, 1.02)	0.01
Year:West	**1.02 (1.01, 1.05)**	**0.01**

Note. Bold is statistically significant (P < 0.05).

## Discussion

Prior analysis of sterile site MSSA cultures within VHA inpatient settings indicated an increase in potential ST398 MSSA from 2003 to 2010, with prevalence plateauing from 2010 to 2014 around 15%.^9^ In this study which includes data through 2019, similar prevalence is observed in cultures from all sites obtained in VHA outpatient settings. This suggests that potential ST398 MSSA has established stable levels of circulation in the US population.

While prevalence over time is relatively static, regional differences persist. Potential ST398 MSSA prevalence remained highest in outpatients residing in the Northeast, consistent with earlier studies that documented early detection of ST398 MSSA in NYC. Significant increases in potential ST398 MSSA are also observed in outpatients residing in the Western region of the United States. Long-distance spread and intense short-distance circulation of ST398 MSSA have been documented using phylogeographic analysis, pointing to migration routes and human circulation as responsible for the diffusion and establishment of this novel type.^4^

Understanding these geographic differences in potential ST398 prevalence, and its associated phenotypic profile, has important implications for empiric therapy, particularly in outpatient settings. In the Northeast, over 20% of the MSSA infections observed in outpatient settings were resistant to clindamycin and erythromycin, and the rising rates of resistance may influence clinicians in that region to change their empiric prescribing patterns based on their risk tolerance. At the same time, clinicians in the West need to be aware of the novel resistance profile that is slowly increasing in prevalence among their patients. While beta-lactam agents (e.g., first-generation cephalosporins) are generally preferred options for MSSA, most empiric therapy decisions, particularly in outpatient settings, are made without susceptibility profile information. As such, epidemiologic trends of phenotypic change can be useful information to guide clinicians’ prescribing patterns.

Sample collection was limited to the end of 2019 to avoid COVID pandemic confounding factors such as decreased outpatient visits and increased antimicrobial usage. Future work will explore potential ST398 MSSA prevalence to understand if spatiotemporal patterns have changed in VHA populations following the pandemic.

Though its patient population is older with higher male proportions than the rest of the US population, the VHA represents one of the only sources for nationwide retrospective phenotypic analysis of bacterial isolates in the United States. While the phenotypic profile used in this study may incorrectly identify ST398 MSSA, it is still useful to observe spatiotemporal trends in resistance profiles. Moreover, many studies focus on MRSA or invasive infections rather than more frequently experienced outpatient illnesses. Understanding the resistance patterns and associated genotypes occurring in MSSA and the community rather than in healthcare settings is an important indicator of the successful spread and persistence of novel *S. aureus* types in the non-hospitalized population.
